# Lipid Modifications of Sonic Hedgehog Ligand Dictate Cellular Reception and Signal Response

**DOI:** 10.1371/journal.pone.0021353

**Published:** 2011-07-01

**Authors:** Vandana K. Grover, J. Gerardo Valadez, Aaron B. Bowman, Michael K. Cooper

**Affiliations:** 1 Department of Neurology, Vanderbilt University Medical Center, Nashville, Tennessee, United States of America; 2 Vanderbilt Brain Institute, Vanderbilt University Medical Center, Nashville, Tennessee, United States of America; 3 Vanderbilt Kennedy Center, Vanderbilt University Medical Center, Nashville, Tennessee, United States of America; 4 Vanderbilt-Ingram Cancer Center, Vanderbilt University Medical Center, Nashville, Tennessee, United States of America; 5 Veterans Affairs Tennessee Valley Healthcare System, Nashville, Tennessee, United States of America; Skirball Institute of Biomolecular Medicine - New York University Medical Center, United States of America

## Abstract

**Background:**

Sonic hedgehog (Shh) signaling regulates cell growth during embryonic development, tissue homeostasis and tumorigenesis. Concentration-dependent cellular responses to secreted Shh protein are essential for tissue patterning. Shh ligand is covalently modified by two lipid moieties, cholesterol and palmitate, and their hydrophobic properties are known to govern the cellular release and formation of soluble multimeric Shh complexes. However, the influences of the lipid moieties on cellular reception and signal response are not well understood.

**Methodology/Principal Findings:**

We analyzed fully lipidated Shh and mutant forms to eliminate one or both adducts in NIH3T3 mouse embryonic fibroblasts. Quantitative measurements of recombinant Shh protein concentration, cellular localization, and signaling potency were integrated to determine the contributions of each lipid adduct on ligand cellular localization and signaling potency. We demonstrate that lipid modification is required for cell reception, that either adduct is sufficient to confer cellular association, that the cholesterol adduct anchors ligand to the plasma membrane and that the palmitate adduct augments ligand internalization. We further show that signaling potency correlates directly with cellular concentration of Shh ligand.

**Conclusions/Significance:**

The findings of this study demonstrate that lipid modification of Shh determines cell concentration and potency, revealing complementary functions of hydrophobic modification in morphogen signaling by attenuating cellular release and augmenting reception of Shh protein in target tissues.

## Introduction

The Hedgehog (Hh) family of signaling proteins are secreted from localized sources and elicit concentration-dependent cellular responses to specify tissue pattern during development and homeostasis [1], [2]. Proper Hh ligand distribution and reception are essential for the full repertoire of graded cellular responses and human birth defects and malignancies are attributed to the misregulation of Hh signaling [3], [4], [5], [6]. A unique biochemical property of the secreted Hh signaling domain is covalent modification by cholesterol and palmitate. Following signal sequence cleavage, cholesterol serves as a cofactor in an autocatalytic intramolecular cleavage reaction and remains covalently bound to the carboxy-terminal Gly residue of the newly formed signaling domain [7],[8],[9],[10],[11]. The second hydrophobic modification is catalyzed by the acyl-transferase Skinny hedgehog, which results in the amide linkage of palmitate to the amino-terminal Cys residue of the signaling domain [12], [13], [14].

Hydrophobic modification confers membrane affinity such that the secreted signaling domain is tightly associated with Hh-generating cells [8], [9], [15]. The cellular release of cholesterol modified Hh ligand is regulated by the transmembrane protein Dispatched [16], [17], [18]. Thus, one biological function of the lipid moieties is to restrict the spatial deployment of Hh morphogens. Hh is secreted as multivalent particles [19], [20] whose formation requires the presence of both lipid additions to the signaling domain [19],[21],[22]. Thus, it has been proposed that both lipid moieties are required for long range signaling [19], [21], [22], [23]. Conspicuously, however, lipid modification is not a requisite for high-affinity binding of Hh ligand to Patched1 (Ptc1) and other receptor complex proteins [24], [25], [26], [27], [28].

A clear understanding of how lipid modification influences signal reception has been hampered by conflicting results. Notably, the earliest *in vitro* signaling assays utilized purified Sonic hedgehog (Shh) lacking both cholesterol and palmitate adducts [29] to elicit the full repertoire of graded signaling responses in explanted chick neural plate ectoderm [30], [31]. Conversely, in cell-based assays removal of either of the lipid adducts abolished [21] or greatly diminished signaling [26], [27]. In *Drosophila* and mouse embryos, localized expression of Hh lacking only the palmitoyl moiety decreased long range signaling [14], [21], whereas the localized expression of Hh lacking only cholesterol broadened tissue distribution and range of signaling [9], [16], [32], [33].

A major constraint of *in vivo* model systems to elucidate the influences of the lipid adducts on Hh signaling is the inability to distinguish an effect on tissue distribution, and thus local concentration, from an effect on signal potency. Another limitation centers on tissue-specific differences in sensitivity to Hh signaling [32]. With regard to cell-based assays, we report a profound loss of signal reception sensitivity in cloned and high-passage cell lines used in prior studies [22], [34], [35]. To circumvent some of these limitations, early-passage NIH3T3 fibroblasts and assays with enhanced sensitivity were used to integrate quantitative measurements of Shh concentration, cellular localization, and potency to evaluate the influences of lipid modification on Shh signaling. We demonstrate that the lipid adducts serve critical functions in cellular reception, governance of cell concentration, and signal potency of Shh ligand.

## Materials and Methods

### Preparation of Complementary DNA (cDNA) Constructs

The pRK5-Shh construct was used to express full length mouse Shh. The pRK5-ShhN construct carries an open reading frame truncated after Gly-198 and was used to express Shh lacking cholesterol [31]. To eliminate palmitoylation, PCR site-specific mutagenesis by overlap extension [36] was performed with pRK5-Shh as cDNA template (Platinum® Blue PCR SuperMix, Invitrogen) with the following primers: 1) 5′ -CCC GGG CTG GCC GCT GGG CCC GGC AG- 3′ (mutates Cys-25 to Ala-25; ShhC25A) and 2) 5′-CCC GGG CTG GCC AGT GGG CCC GGC AG- 3′ (mutates Cys-25 to Ser-25; ShhC25S). To eliminate both cholesterol and palmitate modification, the primers listed above were used in conjunction with 5′-GCG GCC AAA TCC GGC GGC TAG GTC GAC TGC-3′ to create a stop codon after Gly-198. All constructs were sequenced for verification (Genepass Inc.).

### Cell Culture and Transfection

NIH3T3 mouse embryonic fibroblasts cultured in 6-well plates were co-transfected (FuGene® 6, Roche) with Shh constructs (Shh, ShhC25A, ShhC25S, ShhN, or ShhNC25A) over a range of 0.06 ng to 1000 ng in two fold increments, pEGFP-C1, a Gli-reporter (pGL3–8xGli-luciferase) [35], [37], pCMV-LacZ, (a transfection control with a 9∶1 ratio of Gli-reporter:LacZ), and variable amounts of empty vector (pcDNA) to normalize the total DNA quantity in each well. Shh constructs were eliminated from the co-transfection mix for controls. Twelve hours later, cells were changed to low-serum medium (0.5% calf serum) and cultured for an additional forty hours [38], [39] (Figure S1). Cells were then processed by flow cytometry, ELISA, or used chemiluminescent signaling assays. Cells from each well (9.6 cm^2^) were harvested and allocated for flow cytometry, Guava EasyCyte and ELISA. All experiments were conducted in parallel with identical culture conditions. The conditioned medium from each well was also collected for ELISA.

In other studies, NIH3T3 cells were transfected with Gli-reporter (pGL3–8xGli-luciferase) and pCMV-LacZ (in a 9∶1 ratio) changed to low-serum medium with exogenous recombinant Shh protein, incubated at 37°C for 42 hours, and then analyzed by ELISA or chemiluminescent signaling assays. For recombinant Shh protein, HEK293T cells were transfected with a Shh expression construct (Shh, ShhC25A, ShhC25S, ShhN, ShhNC25A, or ShhNC25S), and changed to low serum medium. Thirty-six hours later, conditioned medium was filtered and concentrated (Amicon® Ultra-15 centrifugation filter units; Millipore). For control assays, NIH3T3 cells were transfected as above and exposed to conditioned medium from untransfected HEK293T cells.

### Flow Cytometry

NIH3T3 cells were dissociated with trypsin (Invitrogen) and then washed at 4°C with FACS buffer (phosphate-buffered saline, 2% fetal bovine serum, and 0.05% sodium azide). Anti-Shh antibody (5E1, Developmental Studies Hybridoma Bank) was fluorescently conjugated as directed by the manufacturer (Zenon® Alexa Fluor-647; Invitrogen) and cells were then stained with 5E1-Alexa Fluor-647 anti-Shh antibody (8 mg/mL diluted at 1∶10,000), washed with FACS buffer and fixed in 2% paraformaldehyde. To measure total cellular expression levels, cells were permeabilized and fixed (BD Cytofix/Cytoperm) prior to staining. To measure internal expression, cells were first incubated with a saturating level of unlabeled 5E1 antibody (1∶50; Figure S2) for 1 hour, washed with FACS buffer, and then fixed, permeabilized and stained with fluorescent conjugated 5E1 antibody. Samples were run on a 5-laser BD LSRII system (BD Biosciences) and at least 50,000 viable cells were analyzed per sample. Non-viable cells were excluded from analysis based on forward and side scatter profiles as well as 7-aminoactinomycin D (Invitrogen) staining. Data were acquired using FACSDiva (BD Biosciences) and analyzed using FloJo (Treestar, Inc). Both EGFP (excited at 488 nm Argon Laser, and detected with a 505LP mirror and a 530/30 bandpass filter) and Alexa 647 (excited at 633 nm He-Ne Laser, detected with no LP mirror and a 670/14 bandpass filter) signals were analyzed simultaneously in all cells. Mean fluorescence index (MFI) was calculated by multiplying the percentage of positively stained cells by the mean fluorescence intensity (cells with fluorescence intensity greater than 99% of control transfected cells (Figure S3)). For cell counts, NIH3T3 cells were suspended in phosphate-buffered saline (PBS) and analyzed with the Guava EasyCyte as directed by the manufacturer (Guava® ViaCount® Reagent; Guava Technologies).

### Enzyme-linked immunosorbent assay (ELISA)

Culture medium was collected and then NIH3T3 cells were washed three times with PBS and lysed in RIPA Buffer (50 mM Tris-HCl at pH 7.4, 150 mM NaCl, 2 mM EDTA, 0.1% SDS, 1% NP-40) with protease inhibitors (Complete Mini, Roche Applied Science) on a rotator for 4 hours at 4°C. Recombinant Shh protein concentration was measured in the cell lysate and culture medium as instructed by the manufacturer (DuoSet® ELISA Development System, R&D Systems). ELISA was performed on a series of eight, two-fold dilutions with a starting concentration of 1∶1 (reagent diluent:sample). The colorimetric optical density was measured at 450 nm (FLUOstar Omega, BMG Labtech). An internal positive control (purified Shh protein, 0.063 nM) was included in each assay for normalization. Culture medium and cell lysate from untransfected NIH3T3 cells were used as negative controls.

### Shh Signaling Assays

Chemiluminescence (Dual-Light® Luciferase and ß-Galactosidase Reporter Gene Assay System) was measured in lysed (Passive Lysis Buffer; Promega) NIH3T3 cells as directed by the manufacturer (FLUOstar Omega, BMG Labtech) .

### Statistical Analysis & Data Manipulation

The half-maximal excitatory concentrations (EC_50_) were determined by obtaining the non-linear regression (plotted with a 95% confidence interval) of transformed (X = Log(X)) and normalized (highest value set at 100) data. Significance was determined using one-way analysis of variance and a Bonferroni post-test with 99% confidence intervals. All statistical analyses were performed using GraphPad Prism. To determine the cell-associated concentrations of recombinant Shh protein in transfected cells, the total amount of protein measured in the lysate by ELISA was divided by the number of transfected cells (EGFP^+^) for that well.

## Results

### Either lipid adduct is sufficient for cellular association of Shh ligand

**Figure 1:**
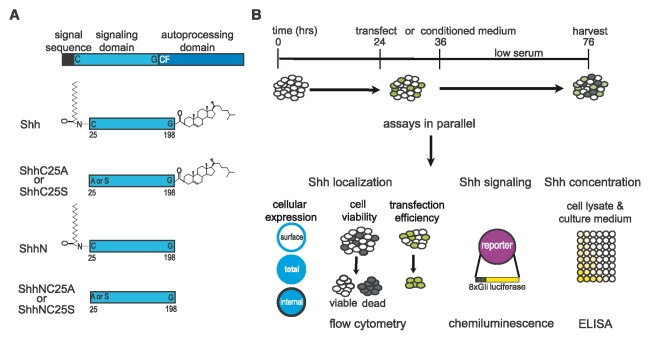
Schematics of experimentation. (A) Shh ligand lipidated with palmitate and cholesterol was generated from a full-length Shh open reading frame. Point mutation of the amino-terminal C25 to either an A or S was introduced to eliminate palmitoylation (ShhC25A, ShhC25S, ShhNC25A and ShhNC25S). Introduction of a stop codon after G198 eliminates cholesterol modification (ShhN, ShhNC25A, and ShhNC25S). (B) Assays of recombinant Shh protein concentration, cellular localization, and signaling were performed on transfected NIH3T3 fibroblasts harvested from the same or parallel wells. ELISA measurements of Shh protein concentration, flow cytometric analysis of cell expression, and chemiluminescence signaling assays in NIH3T3 fibroblasts co-transfected with recombinant Shh, EGFP, Gli-reporter, and LacZ were integrated to determine the contributions of each lipid adduct on Shh secretion, cellular localization, and signaling potency.

**Figure 2:**
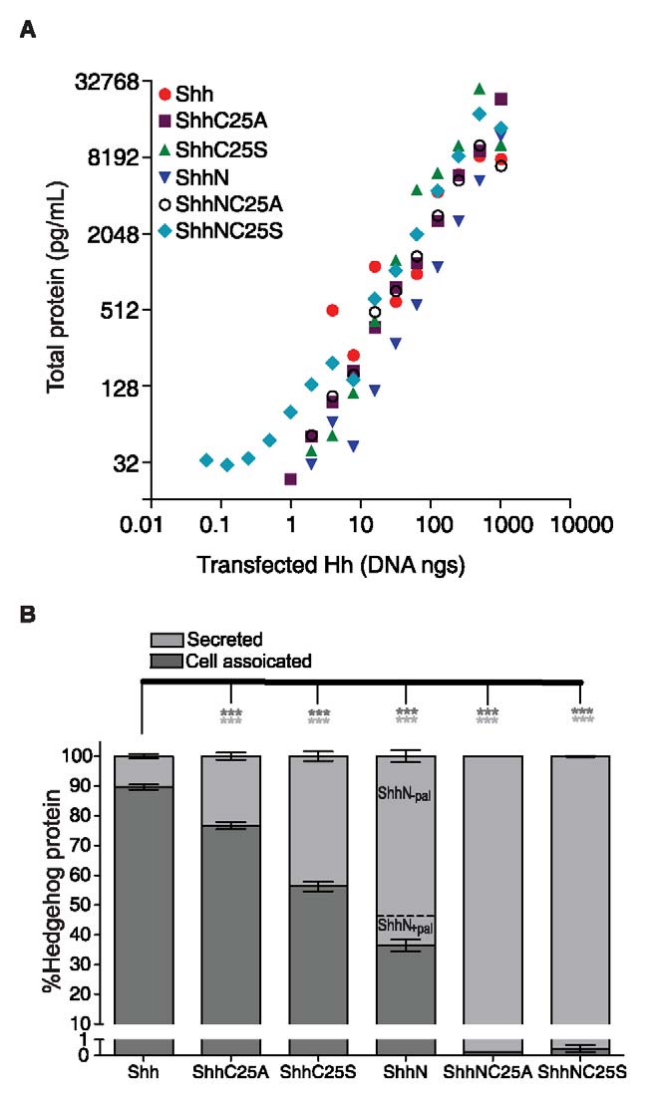
Shh lipid modifications enhance cellular association. (A) Each of the constructs conferred dose dependent and similar expression levels of recombinant Shh protein. Shown are the sums of protein measurements in cell lysate and culture medium from NIH3T3 cells transfected with a range of cDNA for Shh, ShhC25S, ShhN, and ShhNC25S. (B) Precise quantification of recombinant Shh protein concentrations in either the cell lysate or culture medium revealed both cholesterol and palmitate modifications confer cellular association. Shown are the averages of Shh protein measurements from cells transfected with cDNA (62.5 ng to 500 ng in two fold increments). With both lipid adducts, 89.7% of Shh was recovered in the lysate and 10.3% was secreted in the culture medium. Less protein was measured in lysates from cells expressing Shh without a palmitate adduct, (76% for ShhC25A and 56.3% for ShhC25S). Examination of the culture medium from cells transfected with ShhN by HPLC (Figure S1) revealed the presence of two species of protein, one that was palmitoylated (ShhN_+pal_) and one that was not (ShhN_−pal_). Approximately 13% of secreted ShhN was palmitoylated (ShhN_+pal_). The portion of cell-associated ShhN (36.5%) represents the ShhN_+pal_ species because in the absence of either lipid moiety, less than 0.5% of ShhNC25A and ShhNC25S protein was recovered from cell lysate. Protein measurements were performed in replicates of four (± s.e.m.) *** p<0.001 compared to Shh-transfected cells.

The lipid adducts, cholesterol and palmitate, tightly associate Shh ligand with Hh-generating cells [8], [10], [12], [15]. To quantify the contribution of each lipid modification to cellular association, NIH3T3 fibroblasts were co-transfected with expression vectors for recombinant Shh (ranging from 0.06 ng to 1000 ng in two-fold increments) and EGFP, and the concentrations of recombinant Shh protein in culture media and cell lysates were measured by ELISA (Figure 1). All the constructs conferred similar dose-dependent total expression levels of recombinant forms of Shh protein (Figure 2A). The proportions of cell-associated and secreted Shh, however, were significantly altered by lipid modification. Whereas only 89.7% of lipid-modified Shh was cell associated, virtually all (≥99.5%) was released into the culture medium when both lipid modifications were removed (ShhNC25A and ShhNC25S), indicating a strict requirement of lipid modification for cellular association (Figure 2B). Removal of the palmitoyl adduct alone decreased the cellular association of recombinant Shh (76.7% of ShhC25A and 56.3% of ShhC25S; Figure 2B), and the enhanced association of ShhC25A compared to ShhC25S is consistent with the greater hydrophobicity of alanine compared to serine [26]. Removal of the cholesteryl adduct resulted in the retention of 36.5% ShhN (Figure 2B), suggesting a greater contribution of cholesterol to cellular association. The efficiency of ShhN palmitoylation, however, can be reduced in *in vitro* expression systems [12]. Consistent with this observation, we determined that in our transfection system approximately 13% of secreted recombinant ShhN was palmitoylated (ShhN_+pal_), while the remaining secreted protein lacked both cholesteryl and palmitoyl adducts (ShhN_−pal_; Figure S4 and Methods S1). Thirty-six percent of recombinant ShhN protein was measured in cell lysates, and appears to represent palmitoylated protein (ShhN_+pal_) as less than 0.5% of recombinant Shh protein lacking both lipid modifications (ShhNC25A and ShhNC25S) was cell associated (Figure 2B). Therefore, in transfected NIH3T3 fibroblasts, approximately 81% of ShhN_+pal_, was cell associated and 19% was secreted. These data demonstrate that either lipid modification is sufficient to confer cell association, however, ligand quantification by ELISA demonstrates that the palmitoyl adduct provides a greater contribution than previously recognized by Western blotting [15].

### Cholesterol modification is required for cell surface retention and palmitate augments ligand internalization

The contribution of each lipid adduct to cellular association was corroborated by flow cytometric analyses with the anti-ShhN monoclonal antibody 5E1 [40]. The 5E1 antibody blocks Hh signaling, and although 5E1 does not recognize Shh well by Western blotting [24], [41], excellent reactivity (low nanomolar) to the native conformation of Shh has been measured by FACS, ELISA and signaling competition assays [26], [40], [42], [43], [44]. Notably, 5E1 binds a surface domain of Shh formed by non-continuous residues in the Shh linear sequence that is maintained in the absence of either cholesterol or palmitate modification [42], [43], [44]. Staining with Alexa 647-labeled 5E1 antibody was performed to detect recombinant Shh protein expression in transfected cells (GFP-positive) and neighboring cells (GFP-negative). Total cellular expression was measured in permeabilized cells and surface expression was measured in non-permeabilized cells. To detect internal expression levels of recombinant Shh protein, non-permeabilized cells were first incubated with saturating levels of unlabelled 5E1 antibody, permeabilized, and stained with Alexa 647-labeled 5E1 antibody (Figure S2).

**Figure 3:**
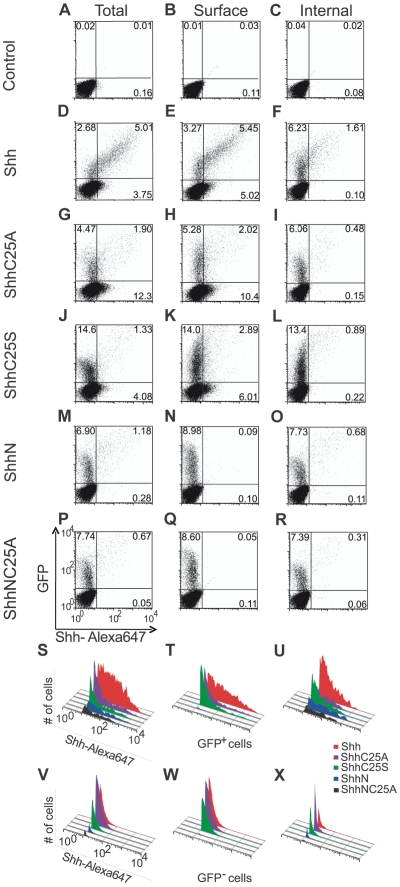
Cholesterol is required for expression of Shh on the cell surface. Shown are flow cytometric data from NIH3T3 fibroblasts co-transfected with recombinant Shh variants (0.125 ng) and EGFP (1000 ng), and stained with 5E1-Alexa 647 conjugated antibody. Total cell staining was measured in permeabilized cells and surface staining was measured in non-permeabilized cells. Internal staining was measured in cells that were pretreated with a saturating level of unlabeled 5E1 antibody prior to permeabilization and staining with 5E1-Alexa 647. Scatter plots (A–R) and histograms (S–X) of 5E1-Alexa 647 staining in transfected (GFP-positive) and untransfected (GFP-negative) cells revealed that cholesteryl modification is required for surface expression (compare E, H & K to N & Q and T to W). In Shh transfected cells (GFP^+^/5E1-Alexa 647^+^) and untransfected cells (GFP^−^/5E1-Alexa 647^+^), the highest ligand staining levels are measured with fully lipidated Shh (D). Removal of either lipid moiety results in decreased cellular retention (G, J, M, P). Shh surface expression (E) is reduced in the absence of palmitate (H, K) and eradicated by the removal of cholesterol (N, Q). Internal expression is also diminished in the absence of either lipid moiety (F, I, L, O, R). Histograms of the number of 5E1-Alexa 647-positive cells within GFP-positive or GFP-negative cells reiterate the above findings (S–X). Experiments were done in replicates of two or more and representative images are shown.

**Figure 4:**
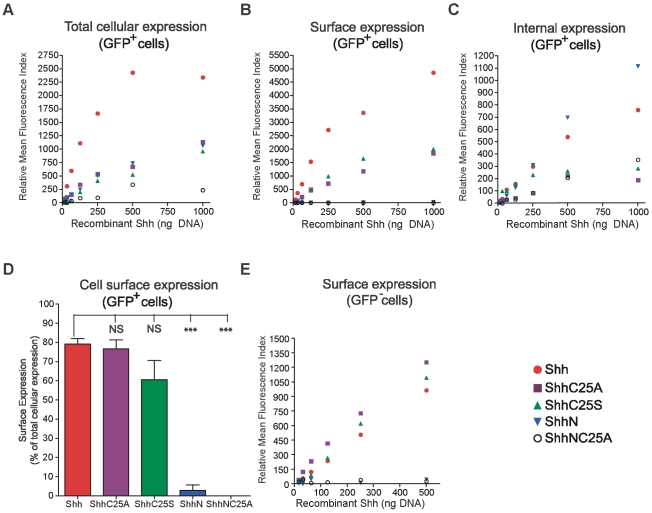
Distinct properties of cholesterol and palmitate modification on cellular localization of Shh ligand. (A–E) Distinct influences of the cholesterol and palmitate modifications on Shh cellular localization were revealed by quantification of mean fluorescence indices (MFI) for 5E1-Alexa 647 staining of NIH3T3 fibroblasts co-transfected with recombinant Shh (0.06 ng to 1000 ng) and EGFP (1050 ng). (A–C) In transfected cells (GFP^+^), removal of both lipid adducts greatly diminished total cell MFI (compare ShhNC25A to Shh in A), and abolished surface expression (compare ShhNC25A in B and C). In ShhN transfected cells, where the vast majority of cell associated recombinant ligand appears to represent a palmitoylated species (ShhN_+pal_), surface expression was greatly diminished and concomitantly internal expression was enriched (compare ShhN in A, B & C). Conversely, following removal of the palmitate alone (ShhC25A & ShhC25S) surface expression was maintained (B) while internal expression levels were reduced to that of recombinant ligand lacking any lipid-modification (compare ShhC25A and ShhC25S to ShhNC25A in C). (D) To quantify the amount of cell-associated Shh ligand that was expressed on the surface, the MFI for internal staining was subtracted from that for total cell staining. Removal of both lipid adducts eliminates surface expression, and only 2.9% of cell-associated ShhN (ShhN_+pal_) was localized to the cell surface. (E) Correspondingly, surface staining with 5E1-Alexa 647 in untransfected cells (GFP^−^) was only measureable for recombinant Shh with cholesterol modification (Shh, ShhC25A, & ShhC25S). Control cells were mock transfected, and MFI is shown relative to control. Experiments were done in replicates of two or more and representative images are shown. NS, not significant (p>0.05); ***, p<0.001 (± s.e.m.) compared to Shh transfected cells.

**Figure 5:**
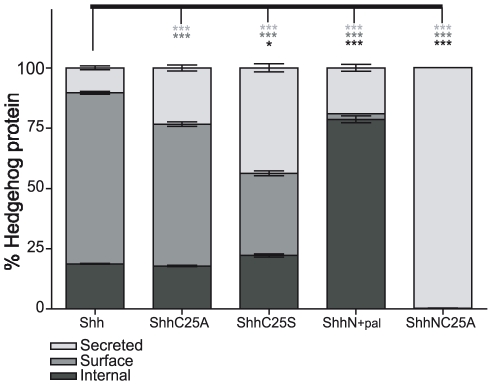
Quantification of the contributions of the lipid moieties to cellular distribution of Shh. Shown are integrated data from ELISA and flow cytometric measurements of recombinant forms of Shh expressed inside cells, on the cell surface, or secreted in the culture medium. The proportion of cell associated and secreted Shh protein for each recombinant expression construct was determined by ELISA measurements of cell lysate and conditioned medium in cells transfected with two fold increments of 62.5–500 ng cDNA (Figure 2B). From the same culture wells, the percentage of Shh protein expressed on the surface and within cells was determined by flow cytometric measurements (Figure 4). To determine the proportion of surface protein expression, the quantity of cell associated Shh was multiplied by the percentage expressed on the cell surface as calculated in Figure 4D. The remaining cell associated Shh protein represents the internal proportion for each recombinant construct. ***, p<0.001; *, p<0.05 (± s.e.m.)

Quantitative flow cytometric analysis of Shh staining within permeabilized GFP-positive cells revealed that both lipid adducts are required to confer the highest degree of cell association (compare Figure 3D to 3P). Correspondingly, we observed a decrement in the percentage of GFP-positive cells that expressed ShhC25A (Figure 3G), ShhC25S (Figure 3J), and ShhN (Figure 3M). These findings were reiterated by examining total cell expression levels over a range of recombinant Shh expression in GFP-positive cells (Figure 4A), and are consistent with an interpretation of the ELISA studies that either lipid moiety can confer cell association. Yet, distinct contributions of the palmitoyl and cholesteryl adducts to steady state cell distribution were revealed. Surface expression was retained in the absence of the palmitoyl adduct (ShhC25A and ShhC25S; Figures 3H, 3K, 3T and 4B). Without the cholesteryl adduct, only 2.9% of total Shh was available on the cell surface while the removal of both lipid adducts entirely eliminated surface expression (Figure 4D). Further, the percentage of cell-associated ligand localized to the surface was not significantly altered by removal of the palmitate (Figure 4D), indicating that the cholesteryl adduct alone is sufficient for cell surface expression (Figure 5). Supporting a requirement of cholesterol for localization to the plasma membrane, its removal (ShhN and ShhNC25A) virtually abolished surface staining (Figures 3N, 3Q, 3T, 4B and 4D). Additionally, the highest internal levels of recombinant Shh were measured within ShhN-transfected cells (Figure 4C). In conjunction with the ELISA data revealing that the ShhN_+pal_ species is cell associated, these data indicate that in the absence of a cholesterol tether to the cell surface the palmitoyl adduct strongly enhances ligand internalization (Figure 5, note that 78.6% of ShhN_+pal_ is expressed inside cells).

Analysis of 5E1 staining in GFP-negative fibroblasts suggests that lipid modification serves analogous functions in receiving cells. In Shh-transfected fibroblasts, 5E1 staining was measured in a significant population of GFP-negative cells (Figure 3D and 3V-3X) and was largely confined to the cell surface (compare 3E to 3F). Conversely, staining in GFP-negative cells was markedly reduced in the absence of both lipid adducts (ShhNC25A; Figure 3P-3R and 3V-3X). Thus, lipid modification strongly enhances ligand association with receiving cells. When solely modified by cholesterol (ShhC25A and ShhC25S), a similar population of GFP-negative cells stained with 5E1 antibody (Figure 3V-3X) and surface localization of ligand predominated (Figure 3G-3L). These results support mathematical modeling studies postulating that cholesterol is the predominant lipid determinant for cell surface association [18]. Notably, surface staining in GFP-negative cells was measurable only for cholesterol modified Shh ligands (Figure 4E). The mean fluorescence indices (MFI) for ShhC25A and ShhC25S surface staining were higher than for Shh in GFP-negative cells as a consequence of the higher levels of cholesterol modified ligand in the culture media under these conditions (Figure 2B). Furthermore, the enhanced surface MFI of ShhC25A with respect to ShhC25S is consistent with the greater hydrophobicity of alanine compared to serine [26].

### Lipid modifications dictate cell concentration and signaling potency of Shh ligand

**Figure 6:**
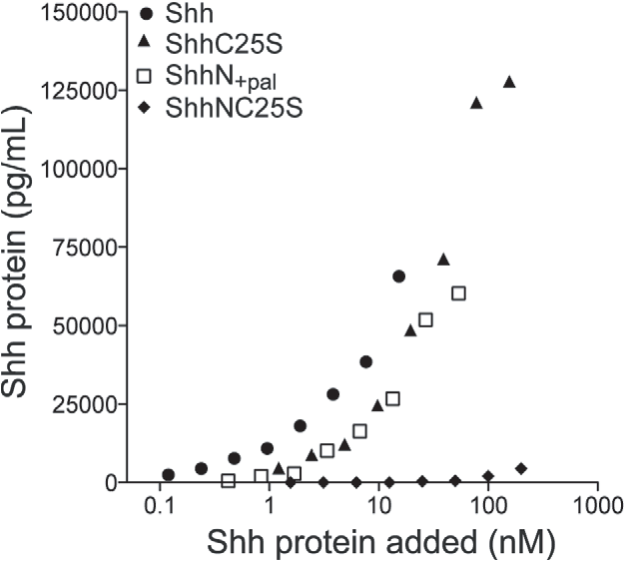
Lipid modification enhances Shh association with receiving cells. NIH3T3 cells were incubated at 37°C for 42 hours with medium containing various concentrations of recombinant Shh protein. After extensive washes, the cells were lysed and analyzed by ELISA. The highest levels of protein were recovered from cell lysates following incubation with Shh-conditioned medium. Recovery of ligand was greatly diminished when both lipid adducts were removed (ShhNC25A), and restored with either cholesteryl (ShhC25S) or palmitoyl (ShhN_+pal_) modification alone. Experiments were performed in replicates of four, and representative data are shown.

The evaluation of 5E1 staining in GFP-negative cells supports a predominant role for cholesterol modification in the association of recombinant Shh ligand with target cells. To directly test the influence of the lipid modifications on ligand association with receiving cells, NIH3T3 fibroblasts were exposed to varying quantities of recombinant Shh protein, and the corresponding amounts of cell-associated ligand was measured by ELISA (Figure 6). By this assay, the highest degrees of cellular association were observed following incubation with fully lipid-modified Shh. Removal of both lipid adducts (ShhNC25S) greatly diminished recovery of ligand in the cell lysate and ShhN_+pal_ and ShhC25S demonstrated equivalent dose-dependent cellular association. Collectively, these data provide direct evidence that the lipid modifications are essential for association with target cells.

**Figure 7:**
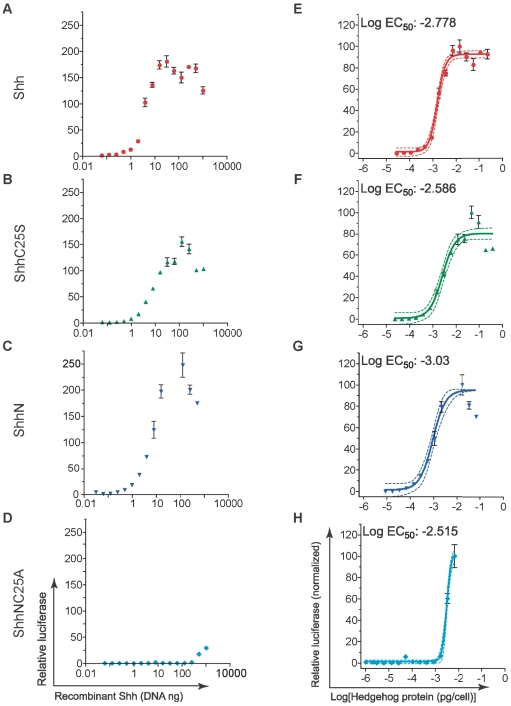
Shh signaling potency is directly related to cellular concentration of ligand. NIH3T3 fibroblasts were co-transfected with expression plasmids for recombinant Shh (Shh, ShhC25S, ShhN, or ShhNC25S), EGFP, Gli-reporter (8xGli-luciferase) and LacZ, changed to low-serum medium for 40 hours, and then analyzed for chemiluminescence. (A–D) Shown are relative luciferase values as a function of transfected recombinant Shh cDNA. ShhNC25A signaling was significantly reduced in comparison to Shh, ShhC25S, and ShhN. (E–H) Analysis of relative luciferase activity as a function of recombinant ligand expressed per transfected cell (pg/cell) revealed that all of recombinant forms of Shh signaled with equal potency. Solid line denotes non-linear regression and the dotted lines denote confidence intervals of 95%. Experiments were performed in replicates of three (± s.e.m.).

**Figure 8:**
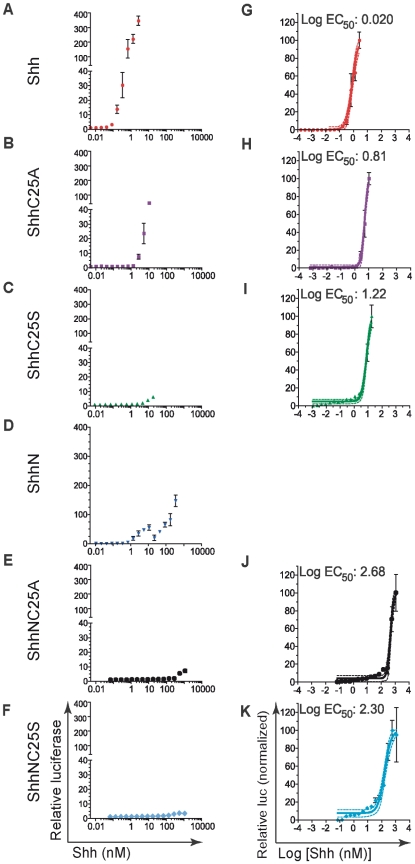
Lipid modification enhances signaling potency of Shh. NIH3T3 fibroblasts were co-transfected with Gli-reporter and LacZ, and changed to low-serum medium containing Shh, ShhC25A, ShhC25S, ShhN, ShhNC25A, or ShhNC25S protein. (A–F) Shown are relative luciferase values as a function of Shh protein (nM) added. (G–K) Normalized relative luciferase measurement of the Log EC_50_ for each variant of recombinant Shh revealed decreased signaling potency following removal of one or both lipid adducts. An EC_50_ could not be calculated for ShhN-conditioned medium because of the complex signaling curve (D). Solid lines denote non-linear regression, and the dotted lines denote confidence intervals of 95%. Experiments were performed in replicates of three (± s.e.m.)

There are conflicting reports regarding the roles of lipid modification and ligand signaling. The signaling potency of Shh ligand devoid of cholesteryl and palmitoyl adducts in C3H10T1/2 cells can be enhanced by the introduction of a wide variety of hydrophobic modifications [26]. Conversely, several studies have reported that removal of either the cholesteryl or palmitoyl adduct abolishes ligand multimerization and signal response in NIH3T3 fibroblasts [19], [21], [22]. We analyzed NIH3T3 cells, which have been used most commonly for Hh signaling assays [19], [21], [22], [35], [39], and found that signal response was greatly reduced in cloned and high-passage lines (Figure S5). In order to determine whether levels of cell-associated ligand correlate with signal response, parallel wells of low-passage and highly responsive NIH3T3 fibroblasts co-transfected with expression vectors for recombinant Shh, GLI-reporter, and EGFP were assayed. In one set of wells, relative GLI-reporter activity was measured and in the other set the concentration of recombinant ligand was determined within GFP-positive cells. When signaling activity was expressed relative to transfected DNA for recombinant Shh, signaling levels were comparable for Shh, ShhC25S, and ShhN and markedly reduced for ShhNC25S (Figure 7A–7D). Yet, when expressed relative to the cellular quantity of ligand, the half-maximal effective concentrations (EC_50_) for each of the recombinant ligands, including ShhNC25S, were nearly identical (Figure 7E–7H and Table 1). The concentrations of recombinant variants of Shh protein recovered from the culture medium was well below the respective EC_50_ calculated in conditioned medium signaling assays, indicating that signaling was mediated by cell-associated ligand in the transfection assays (see Figure 8 and compare Table 1 and Table 2). These data strongly support a direct relationship between signaling potency and cellular concentration of ligand.

**Table 1:** Relative signaling potencies of recombinant forms of Shh ligand.

Recombinant Shh	Cell transfection		Conditioned Media	
	EC_50_ (pg/cell)	Relative to Shh	EC_50_ (nM)	Relative to Shh
Shh	0.0017	1	0.81	1
ShhC25A	0.0077	4.5	5.04	6.2
ShhC25S	0.0026	1.6	7.71	9.5
ShhN	0.00093	0.6	ND	ND
ShhNC25A	ND	ND	311	384
ShhNC25S	0.0031	1.8	104.1	128.5

The half maximal effective concentration (EC_50_) of Shh and recombinant variants lacking one or both lipid modifications was determined in signaling assays with NIH3T3 fibroblasts that were either transfected with recombinant Shh, or to which Shh conditioned medium was added exogenously. ND, not determined.

**Table 2:** Concentration of recombinant Shh in culture medium from signaling assays in transfected NIH3T3 fibroblasts.

Transfected DNA (ng)	500	250	125	62.5
Shh (nM)	0.044	0.035	0.029	0.0085
ShhC25S (nM)	0.59	0.23	0.14	0.11
ShhN (nM)	0.17	0.078	0.03	0.012
ShhNC25S (nM)	0.92	0.43	0.23	0.11

Shown is the amount of recombinant Shh protein measured by ELISA in the culture medium from transfected NIH3T3 fibroblasts. The concentrations of recombinant Shh protein recovered in the culture media are well below the EC_50_ measured for each type of variant when added exogenously to NIH3T3 cells.

When signaling was measured for ligand delivered exogenously to NIH3T3 fibroblasts, the maximal signaling level was highest with Shh and diminished by removal of one or both of the lipid adducts (ShhC25A>ShhN>ShhNC25A; Figure 8A–8E). The complex signaling curve measured for ShhN negated the ability to calculate an EC_50_ in this instance, possibly because of the presence of two species of ligand (ShhN_+pal_ and ShhN_−pal_). However, separation of ShhN_+pal_ from ShhN_−pal_ in ShhN conditioned medium by hydrophobic interaction chromatography revealed that ShhN_+pal_ elution fraction contained the highest signaling potency (Figure S4 and Methods S1). Furthermore, the EC_50_ calculated for Shh, ShhC25A and ShhNC25A correlate directly with measurements of their cell-association properties (Figure 6), supporting the conclusion that the lipid modifications regulate Shh ligand association with receiving cells and dictate signaling potency.

## Discussion

Hedgehog proteins are among several secreted signaling proteins that are covalently modified by lipid moieties (Reviewed in [45]). Hedgehog family members are the only proteins that are known to be modified by cholesterol [11], and this discovery cultivated attention on the influences of lipids on morphogen signaling (Reviewed in [46]). The influences of the lipid modification on ligand release and association with multivalent particles have been well characterized [8], [10], [15], [19], [20]. The roles of the lipid adducts in signal response have been less well defined, however, in part due to constraints of *in vivo* model systems to distinguish effects on tissue distribution from signal potency [21], [23], [32], [33] and of *in vitro* systems with poorly responsive cell lines and qualitative assays of ligand concentration [19], [21], [22]. Utilizing highly responsive NIH3T3 fibroblasts and quantitative assays to integrate measurements of recombinant Shh concentration, cellular localization, and signaling, we demonstrate that the membrane-anchoring properties of cholesterol and palmitate govern the cellular reception of Shh and that signaling potency correlates directly with cellular concentration of Shh ligand. In conjunction with prior studies, these findings illustrate complementary functions of the lipid modifications to attenuate release and enhance reception of Shh signal.

Our studies indicate that either lipid moiety is sufficient to enhance cellular association and increase signaling potency. Cholesterol modification, however, tethers ligand to the plasma membrane while palmitoylation alone is not sufficient for retention on the cell surface. These distinct properties identified in our *in vitro* assays may explain opposing and seemingly puzzling effects on limb patterning observed in prior *in vivo* studies following expression of Shh lacking either the palmitoyl or cholesteryl adduct in the zone of polarizing activity (ZPA). In the mouse limb bud, digit number is reduced by targeted deletion of *Skinny Hedgehog* (*Skn*) to abrogate Shh palmitoylation and interpreted to indicate a requirement for a multimeric Shh protein complex in long range signaling [21]. Long range signaling, however, is enhanced by removal of the Shh processing domain to eliminate cholesterol modification, indicating that cholesterol restricts the long-range movement of Shh protein across the limb bud [33]. In both instances, removal of either cholesterol or palmitate disrupts Shh multimerization [19], [21]. Therefore, our data suggest that the defects in digit specification in *Skn* ^-/-^ limb buds [21] are consistent with Shh protein secreted from the ZPA with reduced potency, resulting from absence of palmitoylation, and restricted long-range movement, due to cholesterylation. Conversely, in limb buds engineered to express ShhN in the ZPA [33], ligand with reduced potency is distributed more broadly due to the absence of cholesterol anchorage to implement low-threshold signaling in the anterior limb bud. Collectively, these observations support a model whereby multimeric or multivalent Shh complexes are not strictly required for Shh signaling, but rather represent a mechanism for delivering soluble and potent lipidated ligand over a range of cells during tissue patterning.

In the absence of Shh ligand, Ptc1 functions to inhibit the pathway by suppressing the activity of the transmembrane protein Smoothened (Smo) [38]. Upon binding with Shh, Ptc1 inactivation allows Smo to initiate signaling [24], [47], [48] through the Gli family of transcription factors (Reviewed in [49]). In vertebrates, primary cilia appear to be the principal site where Shh signaling is regulated by reciprocal subcellular localizations of Ptc1 and Smo [50], [51]. According to this model, Ptc1 localized to the base of primary cilia inhibits the lateral transport of Smo, and binding of Shh to Ptc1 activates signaling by reciprocal movement of Ptc1 out of the cilium and Smo into the cilium [52], [53]. Ptc1 function and the dynamics of its subcellular localization are not fully understood. Sub-stoichiometric levels of Ptc1 suffice to regulate Smo, and the levels of free Ptc1 protein determine the degree of pathway activity as well as the amount of Shh ligand required for pathway stimulation [38]. Ptc1 expression is increased by pathway activation [54]. In NIH3T3 fibroblasts, endogenous Ptc1 is barely detectable by immunofluorescence, and upon pathway activation becomes highly enriched in primary cilia [53]. Against this background, our data may clarify the observation in cell-based assays that lipophilic modification of Shh enhances signaling potency without affecting binding affinity for Ptc1 [12], [26], [27]. Notably, binding assays were performed with cells transfected with a Ptc1 construct truncated at the carboxy-terminal domain to enhance surface expression [12], [26], [27], [48]. We measured a profound reduction in signaling potency and corresponding curtailment in cellular association of unmodified Shh (ShhNC25S and ShhNC25A), suggesting that anchorage of Shh ligand to target cells by lipid modification is critical for access to Ptc1 and other receptor complex proteins. This concept raises the intriguing notion that the lipid modification may also serve to create a reservoir of membrane-associated ligand to maintain durable signaling as cellular levels of Ptc1 are increased. As such, the tethering of Shh ligand to receiving cells could directly influence the temporal and spatial gradients of Shh signaling [55], [56], [57].

## Supporting Information

Figure S1 **Time course of reporter activity in Shh signaling assays.** (A) To determine the linear phase of reporter activity, NIH3T3 fibroblasts were co-transfected with expression plasmids for recombinant Shh (125 ng, 15.63 ng, 1.95 ng, and 0 ng), EGFP, Gli-reporter and LacZ, changed to low serum medium and assayed at varying time points. (B) For signaling assays with conditioned medium, NIH3T3 cells were co-transfected with Gli-reporter and LacZ, changed to low serum medium containing ShhN protein, and then assayed at the indicated time points. Experiments were performed in replicates of three (± s.e.m.).
(EPS)

Figure S2 **Determination of concentration of unlabeled 5E1 antibody required to saturate cell surface staining with labeled 5E1 antibody.** NIH3T3 cells were transfected with Shh cDNA (500 ng) and EGFP (1100 ng), while control cells were transfected with EGFP (1100 ng) alone. The cells were then incubated with varying concentrations of unlabeled 5E1 antibody, fixed, permeabilized, and stained with 5E1 antibody conjugated to Alexa 647 (1∶10000). Saturation, or capping, of surface staining was observed with unlabeled 5E1 antibody over a range of dilutions from 1∶50 to 1∶2000.
(EPS)

Figure S3 **Representative histograms of total, surface, and internal cell staining for Shh.** Shown are histograms for the fluorescent intensity of total (A), surface (B), and internal (C) cell staining for Shh within viable EGFP-positive cells (filled histogram) relative to control cells (unfilled histogram).
(EPS)

Figure S4 **Two distinct species of recombinant protein in ShhN conditioned medium.** (A–B) ShhNC25S and ShhN conditioned media were subjected to hydrophobic interaction chromatography and collected fractions were examined by ELISA. A single peak of ShhNC25S protein was measured in early elution fractions (A). Two peaks of protein were measured for ShhN conditioned medium, one that corresponded to Shh protein devoid of lipid modifications (ShhNC25S), and a second in later elution fractions indicative of greater hydrophobicity. (C–D) ShhN protein in fractions 13 and 22 was quantified by ELISA and assayed for signaling in NIH3T3 fibroblasts transfected with Gli-reporter and LacZ (± s.e.m.).
(EPS)

Figure S5 **Variable signaling responses among lines of NIH3T3 fibroblasts.** (A) NIH3T3 cells stably transfected with Gli-reporter and LacZ (Shh LIGHT Z3 cells) (35) exhibited a dose dependent, but low response to Smoothened Agonist (SAG). (B–C) Three different NIH3T3 cell lines transiently transfected with Gli-reporter and LacZ demonstrated marked differences in signaling competency with pathway activation by co-transfection with Shh (B), the addition of ShhN conditioned medium, or SAG (C). Shh LIGHT Z32 cells and lines 1 and 2 were maintained and passaged over long periods of time. Line 3 was newly purchased from ATCC and expanded in culture over one passage. Fresh aliquots of cryopreserved cells from line 3 were used in all of the experiments in this study. Experiments were performed in replicates of three (± s.d.).
(EPS)

Methods S1 **Column fractionation and analysis.** Conditioned media (DMEM with N2 supplement; Invitrogen) from recombinant ShhN and ShhNC25S transfected and control (untransfected) HEK293T cells were collected, filtered, and then loaded (1 mL loop) on a 15PHE Tricorn 5/100 column (GE Healthcare Life Sciences) equilibrated in buffer (3.9 M ammonium sulfate, 50 mM Tris pH 8). Prior to loading, samples were brought up to a final concentration of 1.5 M ammonium sulfate using a 3.9 M saturated ammonium sulfate in ddH_2_O stock. Bound proteins were eluted after 12 mL wash in equilibration buffer by a 70 mL linear salt gradient beginning with 100% equilibration buffer at fraction 7 to 100% elution buffer (50 mM Tris pH 8) ending at fraction 42. HPLC was performed at 4°C using the Amersham Biosciences ÄKTA Purifier P-900 (GE Healthcare Life Sciences). Fractions were collected in 2 mL increments and analyzed by ELISA or by chemiluminescence signaling assays.
(DOC)
