# Supplementary material for: Lipid Modifications of Sonic Hedgehog Ligand Dictate Cellular Reception and Signal Response
Source: PLoS One. 2011 Jul 1;6(7):e21353. doi: 10.1371/journal.pone.0021353 (PMC3128587; doi:10.1371/journal.pone.0021353)
Supplement: Methods S1 — Column fractionation and analysis. Conditioned media (DMEM with N2 supplement; Invitrogen) from recombinant ShhN and ShhNC25S transfected and control (untransfected) HEK293T cells were collected, filtered, and then loaded (1 mL loop) on a 15PHE Tricorn 5/100 column (GE Healthcare Life Sciences) equilibrated in buffer (3.9 M ammonium sulfate, 50 mM Tris pH 8). Prior to loading, samples were brought up to a final concentration of 1.5 M ammonium sulfate using a 3.9 M saturated ammonium sulfate in ddH2O stock. Bound proteins were eluted after 12 mL wash in equilibration buffer by a 70 mL linear salt gradient beginning with 100% equilibration buffer at fraction 7 to 100% elution buffer (50 mM Tris pH 8) ending at fraction 42. HPLC was performed at 4°C using the Amersham Biosciences ÄKTA Purifier P-900 (GE Healthcare Life Sciences). Fractions were collected in 2 mL increments and analyzed by ELISA or by chemiluminescence signaling assays. (DOC) [file pone.0021353.s006.doc]

**Methods S1**

**Column fractionation and analysis**

Conditioned media (DMEM with N2 supplement; Invitrogen) from recombinant ShhN and ShhNC25S transfected and control (untransfected) HEK293T cells were collected, filtered, and then loaded (1 mL loop) on a 15PHE Tricorn 5/100 column (GE Healthcare Life Sciences) equilibrated in buffer (3.9 M ammonium sulfate, 50 mM Tris pH 8). Prior to loading, samples were brought up to a final concentration of 1.5 M ammonium sulfate using a 3.9 M saturated ammonium sulfate in ddH_2_O stock. Bound proteins were eluted after 12 mL wash in equilibration buffer by a 70 mL linear salt gradient beginning with 100% equilibration buffer at fraction 7 to 100% elution buffer (50 mM Tris pH 8) ending at fraction 42. HPLC was performed at 4 °C using the Amersham Biosciences ÄKTA Purifier P-900 (GE Healthcare Life Sciences). Fractions were collected in 2 mL increments and analyzed by ELISA or by chemiluminescence signaling assays.
